# Maternal *LAMP/p55gagHIV-1* DNA Immunization Induces *In Utero* Priming and a Long-Lasting Immune Response in Vaccinated Neonates

**DOI:** 10.1371/journal.pone.0031608

**Published:** 2012-02-15

**Authors:** Paula Ordonhez Rigato, Milton Maciel, Adriana Letícia Goldoni, Orlando Guerra Piubelli, Noemia Mie Orii, Ernesto Torres Marques, Joseph Thomas August, Alberto José da Silva Duarte, Maria Notomi Sato

**Affiliations:** 1 Laboratory of Dermatology and Immunodeficiencies, LIM-56, Department of Dermatology, Medical School, University of São Paulo, São Paulo, Brazil; 2 Enteric Diseases Department, Infectious Diseases Directorate, Naval Medical Research Center, Silver Spring, Maryland, United States of America; 3 Department of Infectious Diseases and Microbiology, Center for Vaccine Research, Pittsburgh, Pennsylvania, United States of America; 4 Department of Pharmacology and Molecular Sciences, School of Medicine, Johns Hopkins University, Baltimore, Maryland, United States of America; University of Alabama, United States of America

## Abstract

Infants born to HIV-infected mothers are at high risk of becoming infected during gestation or the breastfeeding period. A search is thus warranted for vaccine formulations that will prevent mother-to-child HIV transmission. The *LAMP/gag* DNA chimeric vaccine encodes the HIV-1 p55*gag* fused to the lysosome-associated membrane protein-1 (*LAMP-1*) and has been shown to enhance anti-Gag antibody (Ab) and cellular immune responses in adult and neonatal mice; such a vaccine represents a new concept in antigen presentation. In this study, we evaluated the effect of *LAMP/gag* DNA immunization on neonates either before conception or during pregnancy. *LAMP/gag* immunization of BALB/c mice before conception by the intradermal route led to the transfer of anti-Gag IgG1 Ab through the placenta and via breastfeeding. Furthermore, there were an increased percentage of CD4+CD25+Foxp3+T cells in the spleens of neonates. When offspring were immunized with *LAMP/gag* DNA, the anti-Gag Ab response and the Gag-specific IFN-γ-secreting cells were decreased. Inhibition of anti-Gag Ab production and cellular responses were not observed six months after immunization, indicating that maternal immunization did not interfere with the long-lasting memory response in offspring. Injection of purified IgG in conjunction with *LAMP/gag* DNA immunization decreased humoral and cytotoxic T-cell responses. *LAMP/gag* DNA immunization by intradermal injection prior to conception promoted the transfer of Ab, leading to a diminished response to Gag without interfering with the development of anti-Gag T- and B-cell memory. Finally, we assessed responses after one intravenous injection of *LAMP/gag* DNA during the last five days of pregnancy. The intravenous injection led to *in utero* immunization. In conclusion, DNA vaccine enconding LAMP-1 with Gag and other HIV-1 antigens should be considered in the development of a protective vaccine for the maternal/fetal and newborn periods.

## Introduction

Despite the use of antiretroviral regimens to prevent intrapartum and breast milk HIV transmission [1], mother-to-child transmission (MTCT) of HIV-1 is still the major route of pediatric infection [2]. Effective implementation of drug regimens for long-term prophylaxis is jeopardized by several factors, such as infant toxicity and an inadequate health care infrastructure in some of the most affected countries.

Vertical transmission of HIV-1 depends on the maternal HIV-1 viral load, infant exposure to infected fluids upon delivery and breastfeeding, and the duration and regimen of antiretroviral treatment [3], [4]. Yet, even in the absence of treatment, 55 to 80% of infants exposed to HIV-1 remain uninfected [5].

Children who escape MTCT are again at risk of infection when they become sexually active adolescents. Vaccines that are administered to HIV-1-infected women during the neonatal period or pregnancy and that are able to induce high levels of neutralizing Ab with long-lasting effects are strong candidates for avoiding MTCT. Therefore, studying immunologic interventions, such as maternal and infant vaccine regimens, that would reduce pre- and postnatal HIV transmission remain crucial for protection against HIV infection.

As the newborn immune system is not fully mature, infants are sometimes unable to mount an effective immune response against some pathogens [6], [7]. Passive maternal immunization has been performed to avoid lethal viral and bacterial infections in newborns [6], [8]. However, although maternal Ab represent the major form of protection against diseases in early life, their presence can also interfere with active immunizations and infant immune responses to antigens [9], [6].

There is some evidence that maternal Ab generated after antigen vaccination negatively influence the vaccine response of the infant. However, there is strong evidence that DNA-vaccinated neonates, born from DNA-immunized mothers, can elicit effective immune responses against herpes simplex virus and malaria protozoa even in the presence of maternal Ab [10], [11]. The use of DNA vaccines as a strategy to avoid the inhibitory effects of maternal Ab is based on the fact that the antigen encoded by the vaccine DNA is not exposed to maternal Ab; antigen presentation is carried out by the MHC class I and II molecules of cells that take up DNA [8]. Even if part of the DNA product is secreted and neutralized by maternal Abs, the remaining product will be processed and presented as an endogenous antigen.

Our group has previously studied the *LAMP/HIV-1-gag* chimeric DNA vaccine in neonate models [12], [13]. The lysosome-associated membrane protein-1 (LAMP-1) is highly expressed by all nucleated cells and is found in the MHC class II-enriched intracellular compartments (MIICs) that are associated with antigen presentation [14]. Intradermal delivery of the *LAMP/HIV-1-gag* DNA vaccine has been shown to (i) elicit enhanced Ab production, (ii) amplify the anti- Gag CD4+, CD8+ T and B cells responses, and (iii) prolong immunological memory to Gag epitopes in adult mouse and monkey experimental models [14], [15], [16], [12]. Recently, we have shown that mucosal and intradermal immunization of neonates with the *LAMP/HIV-1-gag* (*LAMP/gag*) construct correlates with strong anti-Gag cellular and humoral immune responses [12], [13].

The rationale for using the *LAMP/gag* DNA strategy in maternal immunization was to verify whether subcutaneous immunization prior to conception could improve the newborn vaccine response by Ab transfer and allow the generation of immunological memory on offspring. Moreover, pregnant mice were also immunized by intravenous route to check whether this change in the maternal immunization protocol could prime the offspring *in utero* and prevent the inhibitory effects of maternal Ab. In fact, there have been reports showing that intravenous DNA injection of pregnant mice can prime the fetal immune system [17], [18]. Furthermore, understanding the strong relationship between fetal and maternal immune systems is essential for developing strategies of maternal vaccination that avoid neonatal antigen tolerance.

In the present work, we evaluated the effect of the *LAMP/gag* DNA vaccine which was administered to mice either before or during pregnancy on the neonatal immune response. The immunization before conception led to a robust transmission of maternal Ab to offspring, which lasted up to 2 months and did not interfere with the long-lasting cellular immune response after neonatal vaccination. Maternal vaccination via the intravenous route with the chimeric vaccine did not raise Ab and led to *in utero* priming of both maternal and fetal immune systems.

## Results

### *LAMP/gag* DNA immunization before conception induces anti-Gag IgG Abs transference through the transplacental and breastfeeding routes to offspring

Transfer of maternal Ab is essential for protecting newborns against infectious agents; maternal vaccination can either prime the neonatal immune system or inhibit the neonatal vaccine response [9]. To evaluate the mechanisms triggered by maternal vaccination with *LAMP/gag* DNA, we first assessed the contribution of IgG transfer to offspring by the transplacental and breastfeeding routes. Female BALB/c mice were immunized and boosted after 20 days by intradermal route with 50 µg *LAMP/gag*, *gag*, or *LAMP* plasmid DNA. The immunized mice were mated and maternal serum, amniotic fluid, and milk were collected. Fetal serum samples were collected by caesarean section, and serum samples from offspring were collected at different ages.

**Figure 1:**
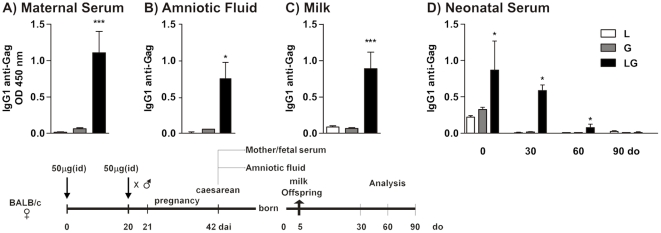
Transfer of high levels of anti-Gag IgG1 Abs from *Lamp/gag* DNA-immunized mothers to offspring through transplacental and breastfeeding routes. Female BALB/c mice were primed and boosted with 50 µg of *Lamp* (L), *gag* (G) or *Lamp/gag* (LG) plasmid DNA and mated one day after the boost. (A) Maternal serum and (B) amniotic fluid were obtained by caesarean section from full-term pregnant mice. (C) Breast-milk samples (1∶20) were collected on day 5 after delivery and (D) fetal serum from 0-, 30-, 60- and 90-d-o offspring were evaluated by ELISA using HIV-1 lysates. The results from 4–6 animals per group were expressed as the mean ± SEM. Schematic diagram of the immunization protocol is shown. *p≤0.05 and ***p<0.001 when compared to *gag*.

We have previously demonstrated that immunization with *LAMP/gag* DNA preferentially induces production of the IgG1 subclass of Abs in adult and neonate mice [12]. Figure 1A shows that the *LAMP/gag*-immunized mothers showed high levels of anti-Gag IgG1 Abs which is also detected in the amniotic fluid (Figure 1B), neonatal serum (fetal serum, day 0, Figure 1D), and milk (Figure 1C). In fact, analysis of neonatal serum showed that there were significantly higher levels of anti-Gag IgG1 Ab in newborns from *LAMP/gag*–immunized mothers than in newborns from *gag*–immunized mothers (Figure 1D). Maternal anti-Gag IgG1 Abs were detected in the serum of offspring from *LAMP/gag*-immunized mothers for up to one month after birth. Anti-Gag IgG2a and IgA Abs were also detected at low levels (Figure S1). Taken together, these results indicated that maternal immunization with *LAMP/gag* DNA before conception promoted a robust passive transfer of anti-Gag IgG Abs through the transplacental and breastfeeding routes both before and after birth.

### Effect of maternal DNA immunization on the vaccine response of offspring

To investigate the effect of maternal immunization with *LAMP/gag* DNA on the offspring immune response, adult female mice were immunized twice with 50 µg DNA at a 20–day interval according to the prime–boost protocol. Mice were mated one day after the boost. Seven–and 25–day old (d-o) offspring were immunized with 5 µg DNA vaccine and evaluated after 30 and 60 days for anti-Gag IgG Ab production. The frequency of IFN-γ-secreting cells stimulated by Gag peptides was evaluated after 60 days. Females and offspring either non-immunized or immunized with the *gag* or *LAMP* DNA vaccine before mating were used as controls.

**Figure 2:**
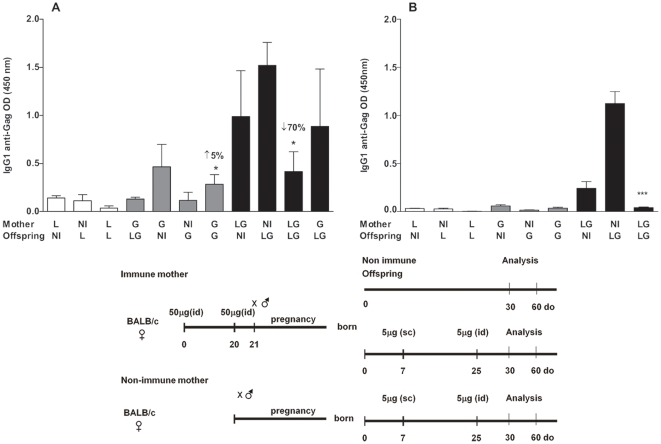
Maternal immunization with the *Lamp/gag* DNA vaccine decreases anti-Gag IgG1 Ab production in immunized offspring. Female mice were immunized, and 20 days later, they were boosted with 50 µg *Lamp* (L), *gag* (G), or *Lamp/gag* (LG) plasmid DNA and mated one day after the boost. Offspring were either non-immunized or immunized with 5 µg of the same vaccine DNA at 7- and 25-d-o. Offspring serum samples were collected at (A) 30- and (B) 60-d-o (3–8 animals per group). Anti-Gag IgG1 was determined by ELISA using HIV-1 lysates. Bars represent the mean ± SEM. Schematic diagram of the immunization protocols are shown. *p≤0.05 and ***p<0.001 when compared to the respective control offspring from non-immunized (NI) mothers.

Figure 2 shows that maternal immunization with *LAMP/gag*DNA promoted the transfer of anti-Gag IgG1 Abs that lasted up to 30 days. *LAMP/gag*–immunized neonates from *LAMP/gag*–immunized mothers displayed a 70% reduction in anti-Gag IgG1 (Figure 2A) and anti-Gag IgG2a Ab levels (Figure S1) compared to immunized offspring from non-immune mothers; whereas non-immunized offspring from *gag*–immunized mothers had almost undetectable levels of maternal anti-Gag IgG1 Abs. When offspring of *gag*-immunized mothers were immunized, the detected Ab levels increased to 5% compared to *gag*-immune offspring from non-immune mother (Figure 2A). The control group of females and their respective offspring immunized with pITR-*LAMP* DNA did not show any anti-Gag response.

**Figure 3:**
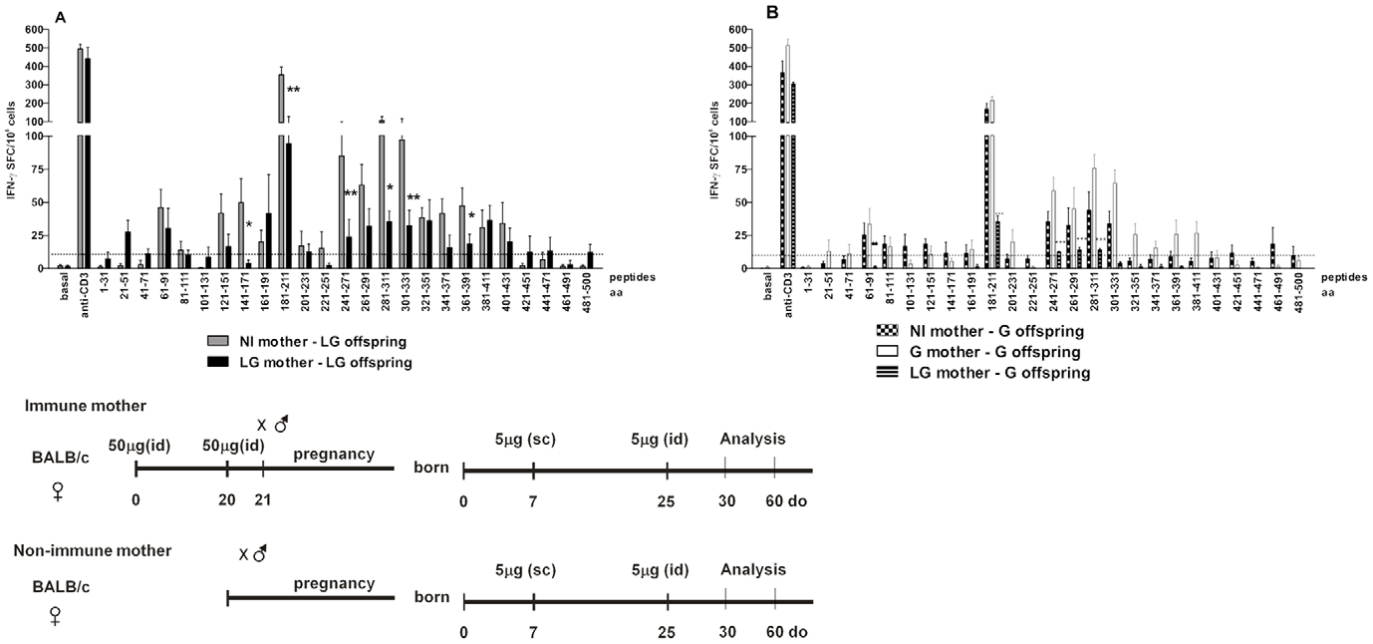
Maternal *Lamp/gag* immunization reduces the IFN-γ response of immunized offspring. Offspring from mothers immunized with *Lamp/gag* (LG) or *gag* (G) were immunized with 5 µg of (A) LG or (B) G DNA, respectively. Spleen cells from 35-d-o offspring were cultured with 25 pooled HIV-1 Gag peptides. The figure represents 4–5 assays per group (2–3 animals per group). Bars show the mean ± SEM. Schematic diagram of the immunization protocols are shown. *p≤0.05 and **p<0.01 when compared with immunized offspring from non-immunized (NI) mothers.

Maternal *LAMP/gag*–immunization led to a decrease in the number of IFN-γ spot–forming cells (SFCs) against Gag peptides in *LAMP/gag*–immunized offspring (Figure 3A). The number of IFN-γ SFCs generated upon stimulation of several peptides, including those containing MHC class I–and II–restricted epitopes, decreased in *LAMP/gag*-immunized offspring from *LAMP/gag*-immunized mothers when compared to *LAMP/gag*-offspring from non-immunized mothers. The immunodominant Gag peptides recognized by MHC class I and II molecules of cells from adult BALB/c mice immunized with *LAMP/gag* and *gag* DNA constructs were peptide fragments from amino acids (aa)181 to aa 227 (MHC class I), aa 241 to aa 271, and aa 281 to aa 311 (MHC class II) as previously reported [15]. Interestingly, maternal *gag* immunization did not interfere with the cellular response (measured by the number of IFN-γ SFCs) of *gag*-immunized offspring when compared to the control group (Figure 3B). The *LAMP/gag* immunization in turn generated broad epitope recognition in the neonatal period even after *LAMP/gag*-maternal immunization. In fact, when mothers were immunized with the chimeric vaccine and the offspring with the *gag* vaccine, the number of IFN-γ SFCs in the offspring decreased (Figure 3B). This inhibitory effect mediated by *LAMP/gag*-maternal immunization was found to be Gag-specific, as there was no decrease in the IFN-γ response to anti-CD3 stimulation. Furthermore, inhibitory effects after maternal *LAMP/gag* immunization were also confirmed when offspring were immunized with 1 µg *LAMP/gag* DNA vaccine (Figure S2).

**Figure 4:**
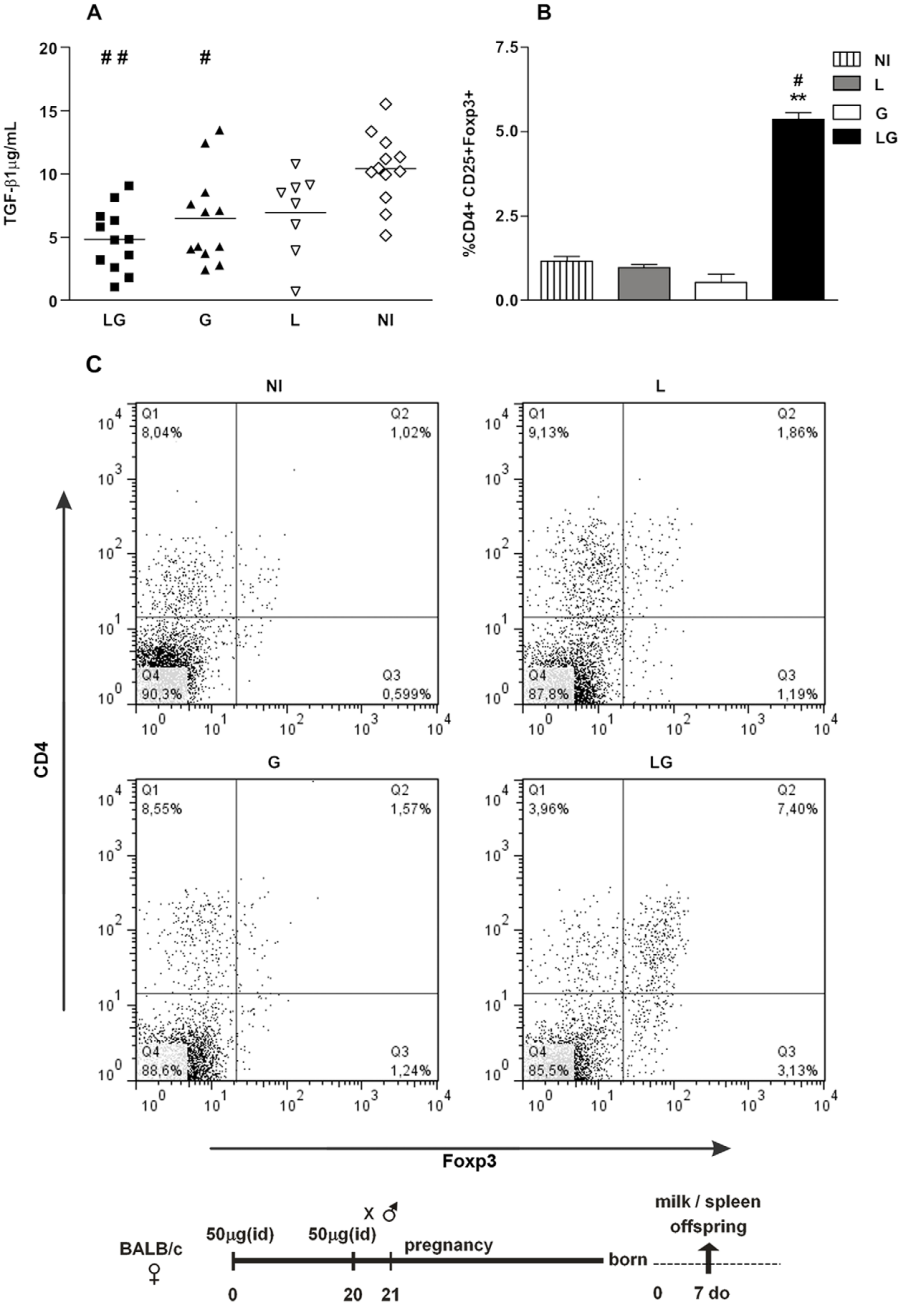
Effect of maternal DNA immunization on TGF-β1 levels in milk and on the generation of CD4+CD25+ FoxP3+ T cells in offspring. Female mice were primed and boosted with 50 µg of *Lamp/gag* (LG), *gag* (G), or *Lamp* (L) plasmid DNA and mated one day after the boost. (A) Breast-milk samples collected 7 days after delivery; (B) percentages of CD4+CD25+Foxp3+ T cells in offspring splenocytes at 7 d-o; (C) CD4+CD25+FoxP3+ T cells in individual offspring at 7 d-o, non-immunized (NI), or immunized with L, G or LG. The results of 8–12 animals per group are expressed as the mean ± SEM. Schematic diagram of the immunization protocol is shown. #p≤0.05 and ##p<0.01 when compared with the non-immunized (NI) group; **p<0.01 when compared with the *gag*-immunized group.

Due to the inhibitory effect of maternal *LAMP/gag* immunization on the offspring vaccine response, we also determined the level of TGF-β1 in milk. TGF-β is one of the important factors involved in the generation of T regulatory (T reg) cells, which contribute to the control of immune responses to vaccines. TGF-β1 levels were evaluated in milk samples that were taken from immune or non-immune mothers 7 days postpartum. Curiously, mothers immunized with either *LAMP/gag* or *gag* DNA showed reduced TGF-β1 levels in their milk 7 days after delivery when compared to non-immunized mothers (Figure 4A).

We next evaluated whether maternal immunization with *LAMP/gag* DNA could interfere with the frequency of T reg cells in the offspring. We quantified the CD4+CD25+Foxp3+ T cells in spleen samples of 7-d-o offspring from immune or non-immune mothers (Figure 4B). Surprisingly, only maternal immunization with *LAMP/gag* DNA augmented the percentage of CD4+CD25+Foxp3+ T cells in comparison to the control offspring.

Therefore, maternal immunization with the chimeric *LAMP/gag* DNA down-modulated the immune response generated in immunized offspring, which could be related to the high levels of anti-Gag maternal Abs present in the offspring as well as to the generation of CD4+CD25+Foxp3+ T cells by the offspring.

### Effect of IgG treatment on LAMP/gag-immunized offspring

We next evaluated whether maternal anti-Gag IgG Abs could be involved in the decreased humoral and cellular anti-Gag immune responses in *LAMP/gag*-immunized offspring. Purified IgG from either non-immune or *LAMP/gag* DNA-immunized mice was injected into 7–, 13–, and 25–d-o offspring from non-immunized mothers. Mice were then immunized at 7–and 25–d-o with 5 µg *LAMP/gag* DNA.

**Figure 5:**
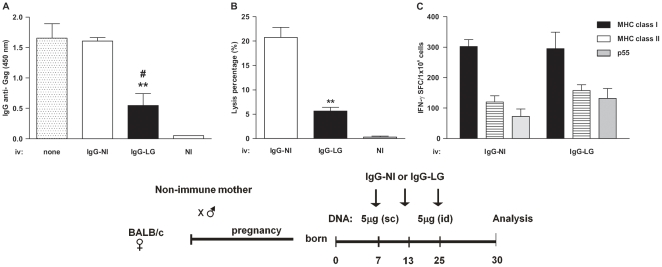
Transfer of IgG from immune mice decreases CTL numbers and anti-Gag IgG Abs in offspring immunized with *LAMP/gag*. Offspring from non-immunized mothers were immunized at 7- and 25-d-o with 5 µg of *LAMP/gag* (LG) and treated with IgG from non-immune (IgG-NI) or LG (IgG-LG)-immunized mice at 7-, 13-, and 25-d-o or without IgG treatment (none). (A) Offspring serum samples from 35-d-o mice (n = 6 per group) were evaluated for anti-Gag IgG Ab levels by ELISA using HIV-1 lysates. (B) *In vivo* T-cell cytotoxicity was evaluated ten days after LG immunization. Mice were IV injected with target cells from NI mice stained with low and high concentrations of CFSE. The CFSE-high target cells were pulsed with a class I immunodominant peptide (AMQMLKETI), and after 18 h, spleen cells were evaluated. (C) IFN-γ SFCs from 35-d-o offspring (n = 3 per group) were evaluated after stimulation with Gag MHC class I and II epitopes or recombinant p55 of HIV-1. Bars represent the mean ± SEM. Schematic diagram of the immunization protocol is shown. **p≤0.01 compared with offspring treated with IgG from NI mice; #p≤0.05 compared with offspring without IgG treatment.

Injection of IgG from immune mice (IgG-LG) led to a significant decrease in the anti-Gag IgG Ab response after offspring immunization (Figure 5A). There was also a decrease in the cytotoxic T cell response towards the H2-K^d^ immunodominant epitope *in vivo* (Figure 5B) when compared to immunized offspring treated with IgG from non-immune mice (IgG-NI). The decreased Ab response that occurred when IgG was injected from immune mice suggested that Ag-specific Abs might inhibit the *LAMP/gag* vaccine response in offspring by forming a complex between Ag and IgG. However, treatment with IgG did not alter the number of IFN-γ SFCs that were generated in response to MHC class I or class II epitopes (Figure 5C). These results showed that treatment with IgG from *LAMP/gag*-vaccinated mice decreased the humoral and CTL responses in immunized neonates, whereas other immune cell functions were not affected.

### Effect of *LAMP/gag* DNA maternal immunization on later T and B cell immune responses on offspring

**Figure 6:**
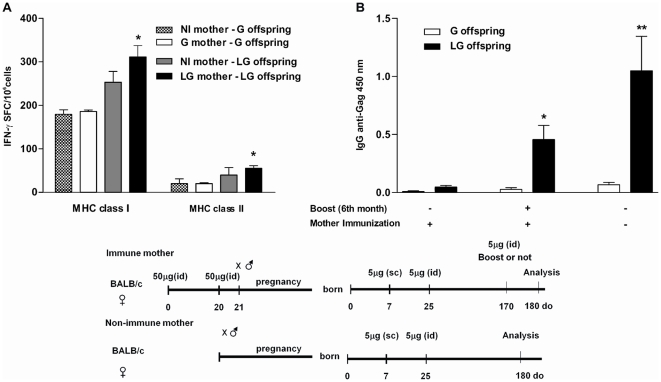
Maternal *LAMP/gag* immunization does not interfere with long-lasting IFN-γ and B-cell responses in immunized offspring. Offspring from non-immune (NI) or immune (I) mothers were immunized at 7- and 25-d-o with 5 µg of either *Lamp/gag* (LG) or *gag* (G) DNA. Some neonates groups from I or NI mothers received a 3^rd^ boost at 6-months-old. (A) IFN-γ SFCs from 6-month-old offspring after stimulation with MHC H-2^d^ class I- and II-restricted pools of Gag peptides from HIV-1. The figure represents 4–5 assays per group (2–3 animals per group); *p≤0.05 when compared with immune offspring from NI mothers. (B) Serum from offspring was collected when they were 6 months old (6 animals per group), and anti-Gag IgG was evaluated by ELISA using HIV-1 lysates. One group received a boost 10 days before analysis. Bars represent the mean ±SEM. *p≤0.05 when compared with immune offspring from NI mothers. Schematic diagram of the immunization protocols are shown **p<0.01 when compared with *gag*-immunized offspring.

Although maternal immunization with *LAMP/gag* DNA inhibits the humoral and cellular immune responses in offspring, it is unknown whether maternal immunization affects the late immune response. The humoral and cellular responses were thus evaluated in 6-month-old offspring from immunized or non-immunized mother. Figure 6A shows that maternal immunization had no negative effect on IFN-γ producing cells in the *LAMP/gag*–immunized offspring for up to 6 months when compared to the control group (LG offspring – NI mothers). Similarly, as expected, maternal immunization with *gag* DNA did not interfere with the long-lasting IFN-γ response in offspring. However, an anti-Gag IgG Ab response was detected only in 6-month-old offspring immunized with *LAMP/gag* DNA following an immunization boost probably due to the high CD4 T cell augmented response given by the chimeric vaccine (Figure 6B). After the 6-month LG DNA boost, the immunized offspring from immune mother showed similar levels of Anti-Gag IgG levels than the immunized offspring from non-immune mother (Figure 6B). These results indicated that although maternal immunization with chimeric *LAMP/gag* DNA affected the early response (10-days after the second boost), it did not impair the late T- and B-cell response (6 months after vaccination).

### Intravenous injection of *LAMP/gag* DNA vaccine during pregnancy primes the fetal immune system

We also investigated whether it was possible to prime the fetus immune system with the chimeric DNA *Lamp/Gag* or conventional (Gag) DNA vaccine by immunizing pregnant mice. We designed a second protocol aiming at (i) decreasing the maternal Ab levels, favoring the priming of the immune system of both mother and offspring, (ii) priming the offspring during the fetal period and (iii) avoiding the presence of high maternal Ab levels transferred to the offspring during their priming with the DNA vaccine. As it is well known that intravenous (IV) delivery is not an immunogenic route, and considering the role of the chimeric *LAMP/gag* DNA on antigen presentation-cell and CD4+ T cell activation, we hypothesized that injecting the DNA vaccines intravenously, seven to five days before delivery, could prime the offspring. We thus injected a 100 µg single dose of the DNA vaccine during the last 7 to 5 days of pregnancy and assessed the effects in the neonate.

**Figure 7:**
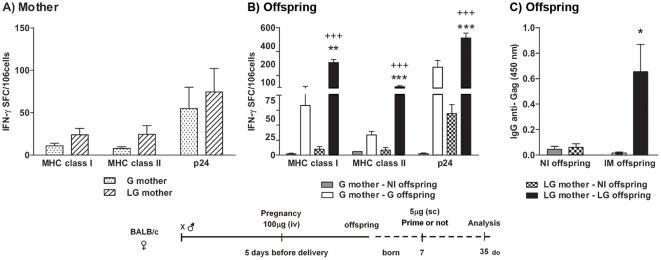
Intravenous DNA immunization during pregnancy primes the fetal immune system. Pregnant mice were IV immunized approximately 15–16 days into gestation with 100 µg of *Lamp/gag* (LG) or *gag* (G) vaccine DNA. Offspring were either immunized (IM) or not (NI) at 7-d-o with 5 µg of the DNA vaccine. (A) Mother: IFN-γ SFCs from spleen cells of immunized mothers 35 days after delivery and (B) Offspring: IFN-γ SFCs from spleen cells of 35-d-o offspring after stimulation with class I and class II Gag peptides and recombinant p24 of HIV-1. The figure represents 2–3 assays per group (2–3 animals per group). (C) Offspring: Serum samples from 35-d-o NI or IM offspring were evaluated by ELISA using HIV-1 lysates for anti-Gag IgG concentrations. Bars represent the mean ± SEM. Schematic diagram of the immunization protocol is shown. *p≤0.05, **p<0.01, and *** p<0.001 when compared with G-offspring from a G-mother; +++p<0.001 when compared with NI offspring from a G-immunized mother.

The results showed that one intravenous injection with 100 µg of *LAMP/gag* or *gag* DNA during pregnancy was able to prime mothers as determined by the induction of IFN-γ SFCs that were generated against the immunodominant MHC class I and II epitopes and the p24 protein (Fig. 7A). We then compared the non-immunized offspring and offspring that were immunized with 5 µg DNA vaccine at 7–d-o. Non-immunized offspring of mothers that received a *LAMP/gag* DNA injection during pregnancy produced IFN-γ SFCs against p24 (average 69.4 SFCs/10^6^MSCs), and this number of IFN-γ SFCs was significantly higher than that of non-immunized offspring of G-immunized mothers (2.54 SFC/10^6^ MSCs). These results strongly suggest that there was *in utero* priming following IV injection of the chimeric *LAMP/gag* DNA vaccine (Figure 7B). In fact, when the 7-d-o offspring from *LAMP/gag*-immunized mothers were then immunized with *LAMP/gag*, the amount of IFN-γ produced in response to the immunodominant MHC class I and II epitopes and to p24 was markedly enhanced compared to offspring immunized with Gag (Figure 7B).

Moreover, non-immunized offspring from mothers immunized at pregnancy had no vertical Ab transfer when mothers were immunized by IV injection (Figure 7C). Therefore, offspring that received just one dose of the *LAMP/gag* vaccine had an increased Ab response when compared to the *gag*-immunized offspring (Figure 7C). Notably, our previous results showed that two vaccine doses are needed and that an individual dose of the *LAMP/gag* vaccine at 7–d-o is not enough to induce anti-Gag Abs [12]. Thus, the current results suggest that there is a recall response upon *in utero* priming in offspring immunized with the *LAMP/gag* vaccine.

In conclusion, our data showed that intravenous administration of the DNA vaccine elicited low numbers of IFN-γ SFCs and primed both maternal and fetal immune systems. These results opened a possibility of priming the fetal immune system *in utero*, with the absence of MatAb, although the intensity of cellular and humoral responses generated by this protocol was lower than that observed with preconceptional immunization of the mother followed by neonatal LG immunization. Then, the preconceptional protocol represent the best approach to transfer Ab and to elicit higher humoral and cellular immune response on immunized neonate, however if we aim to prime as early as possible the fetus immune system and to avoid the effect of MatAb on the offspring vaccine response, we should consider use the intravenous protocol.

## Discussion

Maternal immunization with *LAMP/gag* DNA before conception or during pregnancy was assessed to evaluate the neonatal immune response to the DNA vaccine and the possibility for *in utero* priming, respectively. We have previously shown that administration of the chimeric *LAMP/gag* DNA vaccine at the neonatal stage is highly immunogenic, as it elicits high levels of anti-Gag Abs and elevated cellular immune responses towards the HIV–*gag* antigen when compared to immunization with a DNA construct expressing the Gag antigen only [12], [13]. However, it was unknown whether maternal immunization with the chimeric *LAMP/gag* DNA vaccine could affect the offspring response to the vaccine.

Our results indicated that maternal immunization with the chimeric DNA vaccine by subcutaneous injection before conception promoted passive Ab transfer that caused a transitory inhibition of the offspring response to the construct. Nonetheless, this inhibition did not impair the long-lasting B- and T-cell responses in the offspring. Moreover, the high levels of Ab that were elicited when maternal immunization occurred prior to conception were circumvented when an individual intravenous dose of the *LAMP/gag* DNA was administered during pregnancy. The intravenous route of immunization was less immunogenic than the intradermal route, but it was still able to prime both mother and offspring.

Immunization with the *LAMP/gag* DNA vaccine before conception led to the transfer of high levels of anti-Gag IgG1 Abs through the placental/amniotic and breastfeeding routes. IgG1 Abs are the major subclass of Abs produced by adult and neonate BALB/c mice immunized with *LAMP/gag* DNA [12], and IgG1 Abs were the main Ab subclass that were transmitted to the offspring. Maternal Ab levels lasted up to 60 days in the offspring circulation, indicating that breast milk was an important source of IgG, as weaning takes place at approximately 25 days after birth, and the half-life of IgG is 30 days. The FcRn–IgG Fc receptor, which is expressed in the small intestine of suckling mice and rats, is the main molecule involved in IgG uptake from ingested maternal milk [19]. In mice, the yolk sac endoderm constitutively takes up IgG into the fetal circulation [20]. The *gag* DNA vaccine induced low Ab responses, and consequently, low levels of Ab were transferred to the offspring.

Maternal immunization with *LAMP/gag* DNA decreased the anti-Gag IgG1 and IgG2a Ab levels and the number of anti-Gag IFN-γ SFCs in *LAMP/gag*–immunized offspring when compared to the *LAMP/gag*–immunized offspring from non-immunized the negative impact of *LAMP/gag* maternal immunization on the 35-d-o offspring response to the immunization, it did not hinder the broad T-cell recognition of Gag epitopes in the neonates. It is not clear whether the decreased number in IFN-γ SFC could interfere in the quality of the total anti-Gag immune response; the decrease in the IFN-γ SFC numbers against the class I and II MHC epitopes ― represented by the immunodominant epitopes and not by all of the recognizable epitopes ― by ELISPOT assay do not predict the whole functionality of these Ag-specific T cells. These results should be considered when reviewing how the assays were performed, the ELISPOT assays for determination of IFN-γ SFCs represent an *in vitro* recall response to the stimulating peptides, whereas IgG Ab levels and the cytotoxic assay use *in vivo* or *ex vivo* parameters as a readout.

Nevertheless, six months after neonatal immunization with *LAMP/gag* DNA, no inhibitory effect was detected on the anti-Gag IFN-γ response to class I or II Gag peptides. This result demonstrates that despite the active suppression in early responses, the generation of T-cell memory responses was intact. However, a boost with *LAMP/gag* DNA was necessary to elicit an anti-Gag Ab response in six-month-old offspring. As expected, a B-cell late response was generated only with the *LAMP/gag* immunization and after the 3^rd^ boost, probably due to the higher CD4 T cell activation and T-B cell cooperation.

The presence of maternal anti-Gag Abs in neonates is likely the main inhibitory mechanism for immunizing offspring, as these Ab can form immune complexes with the Gag protein when it is transduced upon *LAMP/gag* DNA immunization. In fact, when neonates were injected with purified IgG from immune mice in conjunction with the *LAMP/gag* DNA immunization, they showed an inhibition of the humoral and *in vivo* cytotoxic T cell responses to the H-2^d^class I immunodominant peptide. Interestingly, the IgG injections did not interfere with the IFN-γ response to the Gag peptides or the p24 protein as compared to the *LAMP/gag*-immunized mice that received IgG from non-immune mice. The *in vivo* cytotoxicity of CD8+ T cells is not dependent on IFN-γ as recently shown in a murine model of prime-boosting using DNA and recombinant adenoviruses against Chagas disease; here, protection against the disease was reduced in IFN-γ^−/−^ mice even in the presence of specific CTLs [21]. However, it is still possible that other regulatory mechanisms independent of maternal Abs contribute to the attenuation of the offspring immune response to the vaccine.

Several mechanisms by which maternal factors influence the immune response in offspring have been described and suggested, including the following: (i) formation of immune complexes with the antigen, which impairs neonatal priming [8]; (ii) anti-idiotypic maternal Ab interaction with immature B and T cells in primary lymphoid organs, or negative or positive modulation of plasma and T cell differentiation [22]; (iii) maternal transfer of cytokines that are able to control cell activation by downregulating the expression of co-stimulatory and activation molecules on dendritic cells and T and B lymphocytes [23], [6]; (iv) interaction of maternal Ab immune complexes with the B-cell inhibitory receptors that mediate negative regulation of B-cell function [23]; and (v) generation of T regulatory cells [6]. Our group has been exploiting the capacity of maternal immunization with allergens to dowmodulate the newborn allergic response [24], [23], [25], [26]. Our findings have shown that allergy prevention in offspring whose mothers were immunized is mainly due to the formation of immunocomplexes with allergens and maternal Abs as well as the upregulation of the inhibitory FcγRIIb receptor in offspring B cells [25], [23].

Notably, one effect of the maternal immunization with *LAMP/gag* was the induction of a high percentage of CD4+CD25+FoxP3+ T cells in 7-d-o neonates despite the reduction of TGF-β1 levels in milk. TGF-β1 is essential for the generation of induced T reg cells [27]; however, it is not clear why only offspring from *LAMP/gag*-immunized mothers showed increased percentages of CD4+CD25+FoxP3+ T cells in the spleen. It is possible that other factors are involved in the modulation of the offspring immune response promoted by the *LAMP/gag* immunization. In fact, it has been shown that T reg cells regulate inflammation during infections, but a high number of T reg cells can suppress the antiviral response [28]. Furthermore, the cells with phenotype of T reg generated after maternal *LAMP/gag* DNA vaccination could function in controlling offspring immune activation after immunization.

There is evidence that to achieve an efficient immune response in offspring, maternal Abs should be directed against epitopes other than neonatal epitopes, thereby avoiding inhibition the offspring response to vaccination [29]. In this case, we should consider the use of different DNA constructs in maternal and offspring immunizations and find a vaccination protocol that is immunogenic in the newborn and can overcome the inhibition caused by maternal Abs. Considering that the chimeric *LAMP/gag* DNA raised Ab titers by 50 to 100-fold and that it was more immunogenic during the neonatal period, we evaluated the effects of IV immunization of pregnant mice with *LAMP/gag* DNA on the immunogenicity of *LAMP/gag*-vaccinated offspring. Maternal immunization was performed close to birth to avoid high levels of Ab transfer to offspring. Our results showed that IV immunization with *LAMP/gag* DNA five to seven days before delivery generated T and B cell responses in mothers and offspring, confirming that is possible to use a DNA vaccine to prime both the fetus *in utero* and the immune system of the mother. Moreover, this approach confirmed that the *LAMP/gag* DNA construct was able to elicit multiple immune responses compared to conventional *gag* DNA immunization, particularly T-cell activation. Despite a lower magnitude of the immune response observed with the intravenous DNA immunization, just one dose of the chimeric vaccine by intravenous injection in the last few days of pregnancy was able to prime both maternal and fetal immune systems. The transfer of plasmid to the fetus through the placenta and amniotic fluids has been a concern, as it may cause tolerance of the offspring immune system towards the plasmid-encoded antigen. The current study and those by others have confirmed that this is not the case [30], [17], [18], [31]. Moreover, use of *LAMP/gag* DNA vaccination during pregnancy could be an advantage by subjecting newborns to fewer doses but enough doses to generate an immune response.

Together, our data showed that the chimeric *LAMP/gag* DNA construct, which targets the Gag antigen to MIIC compartments, more efficiently induced T and B cell responses in mice at various stages in life, compared to the conventional DNA construct expressing *gag* only. Even in the presence of MatAb, the *LAMP/gag* DNA, administered in the neonatal period, elicited broad T cell responses and considerable B cell responses. Furthermore, preconceptional immunization with *LAMP/gag*, inducing MIP-1a secretion (data not show) – natural blocker of infection - and increasing T reg cell numbers in the offspring, could be protective against HIV-1 infection by inhibiting viral entry into target cells and reducing CD4+ T cell activation. Moreover, giving the *LAMP/gag* DNA vaccine during pregnancy primed both maternal and fetal immune systems. Therefore LAMP-1 with other HIV-1 antigens should be considered in the development of a protective vaccine for the maternal/fetal and newborn periods.

## Materials and Methods

### Mouse immunization

8- to 10-week-old BALB/c mice were purchased from CEMIB (UNICAMP, Campinas, São Paulo, Brazil) and bred in our pathogen-free animal facility. The São Paulo University Institutional Animal Care and Use Committee approved the present study.

### Plasmids

A eukaryotic expression plasmid with the fragment of the HIV-1 HXB2 *p55gag* gene, nucleotides 1-1503 (GenBank accession number KO3455), was inserted into the mammalian expression vector, pITR [32], which contains a cytomegalovirus promoter and the ITR sequences from adeno-associated virus flanking the expression elements. The mouse *LAMP-1* gene (GenBank J03881) was cloned into this vector, resulting in the chimeric *LAMP/gag* plasmid with the *p55gag* sequence placed between the *LAMP-1* luminal domain and the transmembrane/cytoplasmic tail as previously described [14]. Plasmid DNA was produced by transforming DH5α *Escherichia coli* (Invitrogen, Carlsbad, CA), and plasmids were purified using an endotoxin-free column (Qiagen Inc., Valencia, CA).

### Immunization protocols

#### Maternal immunization before conception

Female BALB/c mice were immunized (homologous prime-boost) twice by the intradermal route (ID immunized) at a 20-day interval with 50 µg of one of the following plasmid DNAs: pITR-*LAMP/gag* (*LAMP/gag*-LG), pITR-*gag* (*gag*-G), or pITR-*Lamp* (*Lamp*-L). Mice were mated after the boost. Offspring were immunized with the same vaccine as their mothers.

#### Maternal immunization during pregnancy

Pregnant BALB/c mice received 100 µg of the *LAMP/gag* or *gag* DNA vaccine by intravenous (IV) injection 7-5 days before delivery.

#### Neonatal immunization

7- and 25-day-old (d-o) neonates were ID immunized with 1 or 5 µg of *LAMP/gag* (LG), *gag*, or *Lamp* DNA and subjected to analysis at different ages.

#### Passive transfer of IgG from immunized mice

Neonate mice received intraperitoneal (IP) injections at 7-, 13-, and 25-d-o with 100 µg of purified IgG from immune (LG) and non-immune (NI) mice, similar to the DNA immunization protocol. IgG was purified using the Melon Gel IgG Spin Purification kit according to the manufacturer's instructions (Pierce, Rockford, IL) and stored at −70°C until use. IgG measurements were performed by enzyme- linked immunosorbent assay (ELISA).

### Collection of amniotic fluid, fetal and maternal sera and milk

To evaluate vertical transmission of Ab, amniotic fluid and fetal serum samples were obtained by caesarean section of full-term pregnant mice (21 days) and stored at −70°C until use. Mothers were bled at the time of parturition and on day 20 postpartum. Milk samples were obtained from the stomachs of 5- or 7-d-o newborns as previously described [33].

### Ab response

Anti-Gag IgG Ab levels were assessed by ELISA) as previously described [12]. Briefly, 96-well microplates (Greiner, German) were coated with 5 µg/mL HIVIIIB lysate (ABI, Rockville, MD) and incubated overnight at 4°C. After blocking with PBS-0.5% gelatin for 1 h at 37°C, the plates were washed three times with PBS and incubated with serially diluted sample for 2 h at 37°C. After washing with PBS-0.5% Tween, the plates were incubated with biotinylated Ab anti-γ1 (Southern Biotech, Birmingham, Alabama, USA) for 1 h at 37°C followed by the addition of streptavidin peroxidase (Sigma, St. Louis, MO, USA). The reaction was developed with tetramethyl benzidine (Calbiochem Corporation, San Diego, CA, USA), and the absorbance was read at 450 nm in an ELISA microplate reader (Bio-Rad, USA).

### Isolation of mononuclear spleen cells

Spleens were obtained aseptically and mashed through cell strainers (BD-Biosciences, San Diego, CA, USA), and mononuclear spleen cells (MSCs) were isolated after centrifugation in Ficoll-Hypaque solution (Sigma, St. Louis, MO, USA). After two washes, the cell pellet was diluted in RPMI 1640 containing 10% fetal calf serum (FCS, HyClone III, Logan, UT, USA). Viability was greater than 95%.

### Gag-specific IFN-γ ELISPOT assay

Enumeration of IFN-γ–producing splenic T cells was performed by ELISPOT assay (BD-Biosciences) as previously described [12]. Briefly, 96-well microplates with polyvinylidene fluoride membrane support (Millipore, Bedford, MA, USA) were incubated with anti-IFN-γ for 18 h at 4°C, washed and blocked with RPMI containing 10% FCS for 2 h at room temperature. Cells (5×10^5^ cells/well) were plated in the presence of medium alone, hamster monoclonal anti-mouse CD3 (1 µg/mL,BD-Biosciences),10 µg/mL HIV-1 Gag epitope restricted to H-2^d^ MHC class I AMQMLKETINEEAAE_197–211_,10 µg/mL HIV-1 Gag epitope restricted to H-2^d^ MHC class II VDRFYKTLRAEQASQ_297–308_(NIH AIDS Research and Reference Reagent Program), 2 µg/mL p24Gag from HIV-1 (kindly provided by Prof. Luis Carlos Ferreira, Institute of Biomedical Sciences, University of São Paulo), or 10 µg/mL of 25 pools containing five HIV-Gag peptides (15-mers of the HIV-1 HXB2gag peptide), totaling 123 peptides, for 18 h at 37°C in 5% CO_2_. The plates were washed, incubated with biotinylated anti-IFN-γ followed by incubation with avidin-peroxidase, and developed with the 3-amino-9-ethylcarbazole substrate (Calbiochem). Spots were quantified with an ImmunoSpot Imager using the ImmunoSpot 3.2 software (CTL ImmunoSpot® S4 Analyzer, C.T.L., Cleveland, OH, USA). The reaction was considered positive when the number of spot-forming cells (SFCs) was equal to or higher than 10 SFC/10^6^ cells. All results were expressed as the mean number of SFCs per 10^6^ MSCs.

### In vivo analysis of CTL activity

Ten days after immunization with 5 µg of the DNA vaccine, offspring received target cells as previously described with modifications [12]. Briefly, MSCs from non-immunized (NI) BALB/c mice were used as target cells, and these cells were stained with high (10 µM) or low (1 µM) concentrations of carboxyfluorescein succinimidyl ester (CFSE, Molecular Probes, USA) in RPMI medium and incubated for 30 minutes at 37°C in the dark. The population stained with high concentrations of CFSE was pulsed with 2 µg of the class I peptide, AMQMLKETI_197–205_, for one hour at 37°C. Target cells with low and high concentrations of CFSE were washed and mixed at a 1∶1 ratio in PBS. Approximately 3×10^7^ cells in 200 µL of PBS were IV injected into immunized mice (IM). Splenocytes from IM were collected after 18 hours, and transferred target cells were evaluated by flow cytometry based on high and low intensities of CFSE staining. The lysis percentage was determined by the following calculation: percentage of specific lysis = 100−{[(% CFSE^high^ cells IM/% CFSE^low^ cells IM)/(% CFSE^high^ cells NI/% CFSE^low^ cells NI)]×100}.

### Immunophenotyping of CD4+CD25+FoxP3+ T cells

MSCs were incubated with PerCP-conjugated anti-CD4 (BD-Biosciences), PE-conjugated anti-CD25 or isotype control for 30 min at 4°C. After washing, the cells were fixed with 4% paraformaldehyde for 10 min at 4°C. Cells were then incubated with PBS–0.5% saponin (Sigma) and FITC-conjugated anti-FoxP3 (eBioscience, San Diego, CA). After washing, the cells were analyzed by flow cytometry (BD FACSCalibur, Cell Quest PRO). A minimum of 250,000 events was collected from the lymphocyte gate.

### Measurement of TGF-β1 in milk samples

An ELISA for TGF-β1 (Promega, Madison, USA) was performed to measure concentrations in milk samples according to the manufacturer's recommendations. The detection limit of TGF-β1 was 32 pg/mL.

### Statistics

Values for all measurements are expressed as the mean ± SEM. Comparison between two groups was performed by the Mann-Whitney test; differences were considered significant when the p value was <0.05. Three or more groups were analyzed using the Kruskall-Wallis test with Dunn's post-test. The data were analyzed using the GraphPad Prism 3.0 software package (GraphPad, San Diego, California, USA).

## Supporting Information

Figure S1 **Transfer of anti-Gag Ab to offspring.** Female BALB/c mice were primed and boosted with 50 µg of *Lamp* (L), *gag* (G), or *Lamp/gag* (LG) DNA and mated one day after the boost. (A) Serum samples from 0- (fetal), 30-, 60- and 90-d-o offspring were obtained by caesarean section from full-term pregnant mice, and offspring were bled monthly; (B) breast milk samples (1∶20) were obtained 5 days after delivery, and anti-Gag Ab levels were determined by ELISA using HIV-1 lysates. The results of 4–6 animals per group were expressed as the mean ± SEM. *p≤0.05 and **p<0.01 when compared to *Lamp*.
(TIF)

Figure S2 **Maternal** ***Lamp/gag*** **immunization reduces the IFN-γ response in immunized offspring.** Offspring from mothers immunized with either *gag* (G) or *Lamp/gag* (LG) were immunized with 1 µg of (A) LG or (B) G DNA. Spleen cells from 35-d-o offspring were cultured with 25 pooled of HIV-1Gag peptides. The figure represents 4–5 assays per group (2–3 animals per group). Bars represent the mean ± SEM. *p≤0.05 and **p<0.01 when compared with immune offspring from non-immunized (NI) mothers.
(TIF)
