# Supplementary figures and images for: Maternal LAMP/p55gagHIV-1 DNA Immunization Induces In Utero Priming and a Long-Lasting Immune Response in Vaccinated Neonates
Source: PLoS One. 2012 Feb 15;7(2):e31608. doi: 10.1371/journal.pone.0031608 (PMC3280311; doi:10.1371/journal.pone.0031608)

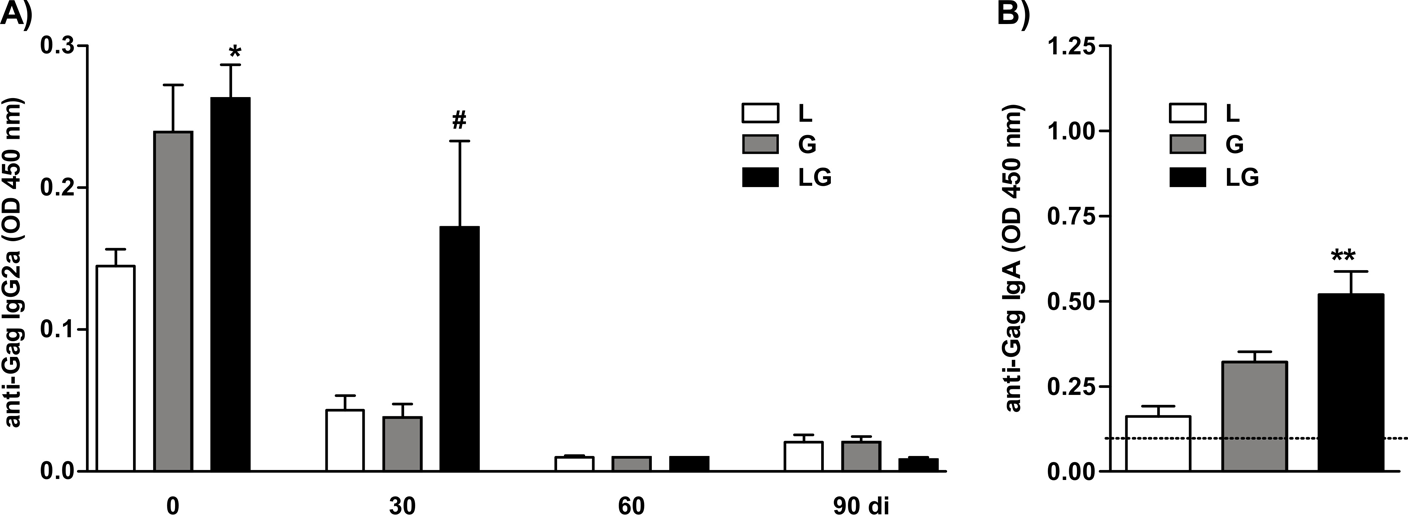

Supplement: Figure S1 — Transfer of anti-Gag Ab to offspring. Female BALB/c mice were primed and boosted with 50 µg of Lamp (L), gag (G), or Lamp/gag (LG) DNA and mated one day after the boost. (A) Serum samples from 0- (fetal), 30-, 60- and 90-d-o offspring were obtained by caesarean section from full-term pregnant mice, and offspring were bled monthly; (B) breast milk samples (1∶20) were obtained 5 days after delivery, and anti-Gag Ab levels were determined by ELISA using HIV-1 lysates. The results of 4–6 animals per group were expressed as the mean ± SEM. *p≤0.05 and **p<0.01 when compared to Lamp. (TIF) [file pone.0031608.s001.tif]

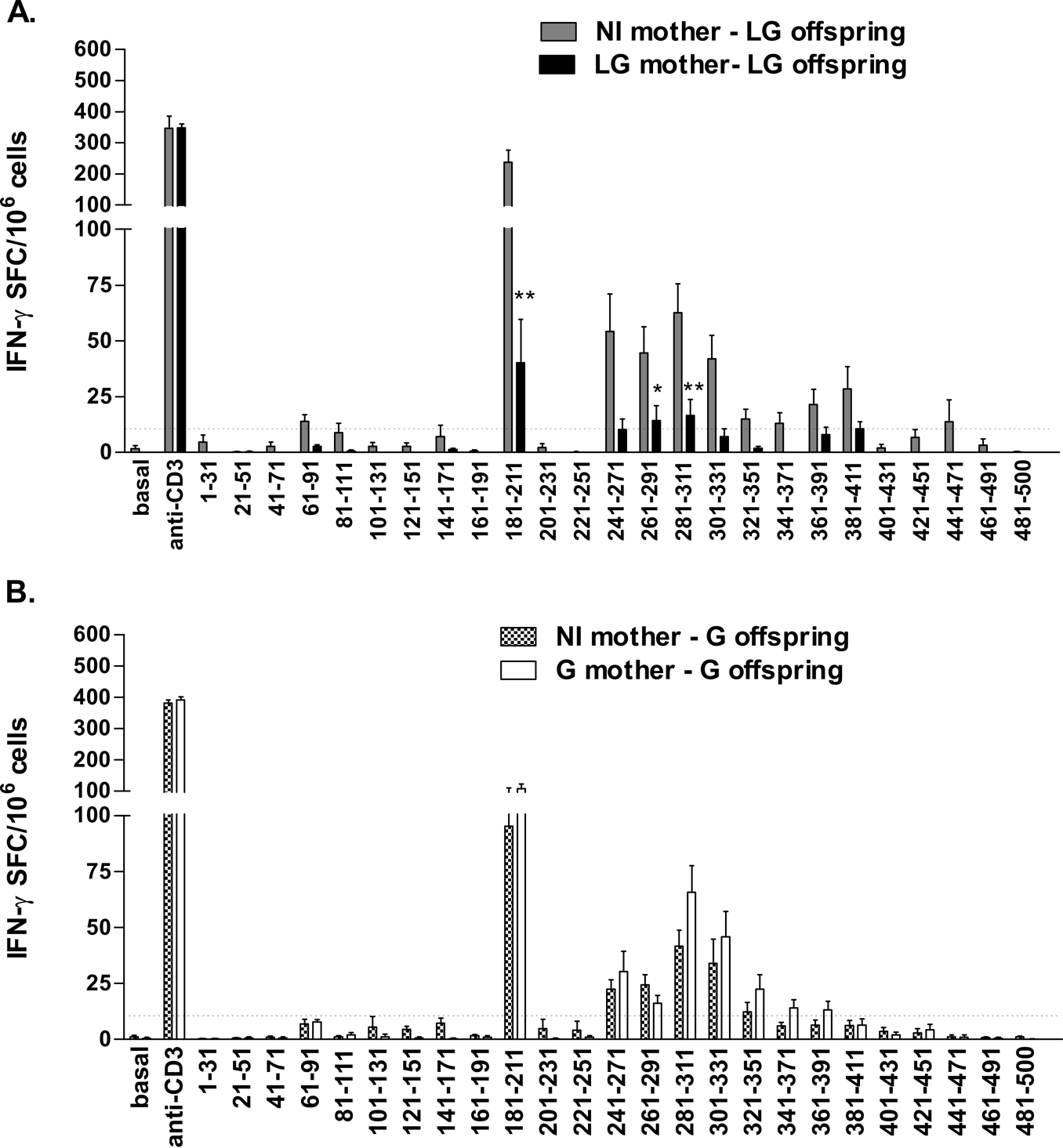

Supplement: Figure S2 — Maternal Lamp/gag immunization reduces the IFN-γ response in immunized offspring. Offspring from mothers immunized with either gag (G) or Lamp/gag (LG) were immunized with 1 µg of (A) LG or (B) G DNA. Spleen cells from 35-d-o offspring were cultured with 25 pooled of HIV-1Gag peptides. The figure represents 4–5 assays per group (2–3 animals per group). Bars represent the mean ± SEM. *p≤0.05 and **p<0.01 when compared with immune offspring from non-immunized (NI) mothers. (TIF) [file pone.0031608.s002.tif]
